# Economic costs of alcohol consumption in Thailand, 2021

**DOI:** 10.1186/s41256-023-00335-w

**Published:** 2023-12-11

**Authors:** Chaisiri Luangsinsiri, Sitaporn Youngkong, Usa Chaikledkaew, Oraluck Pattanaprateep, Montarat Thavorncharoensap

**Affiliations:** 1 Doctor of Philosophy Program in Social, Economic, and Administrative Pharmacy, Department of Pharmacy, Faculty of Pharmacy, Mahidol University, Bangkok, Thailand; 2 Department of Clinical Pharmacy, Faculty of Pharmacy, Srinakharinwirot University, Nakhon Nayok, Thailand; 3 Mahidol University Health Technology Assessment (MUHTA) International Graduate Program, Mahidol University, Bangkok, Thailand; 4 Social and Administrative Pharmacy Excellence Research (SAPER) Unit, Department of Pharmacy, Faculty of Pharmacy, Mahidol University, 447 Sri-Ayudhaya Rd., Rajathewi, Phayathai, Bangkok, 10400 Thailand; 5 Department of Clinical Epidemiology and Biostatistics, Faculty of Medicine Ramathibodi Hospital, Mahidol University, Bangkok, Thailand

**Keywords:** Cost-of-illness, Economic burden, Alcohol, Cost, Drinking

## Abstract

**Background:**

Alcohol is one of the leading risk factors contributing to avoidable economic loss worldwide. Estimates from the economic cost of alcohol consumption studies play an important role in prioritizing healthcare resource use, supporting policy decisions, and justifying budgets for alcohol policy. This study aimed to estimate the economic burden associated with alcohol consumption in Thailand in 2021.

**Methods:**

Prevalence-based cost of illness methodology was employed. The following costs were included in the analysis: healthcare costs; cost of law enforcement; cost of property damage due to road traffic accidents; cost of premature mortality; and cost of absenteeism from out-patient hospital visits and hospitalization. Human capital approach was adopted. All costs were presented in Thai baht, 2021.

**Results:**

Alcohol consumption incurred a total estimated economic cost of 165,450.5 million baht, equivalent to 1.02% of Gross Domestic Product (GDP) and 2500 baht per capita. Cost of premature mortality was estimated at 157,918.7 million baht and accounted for the largest proportion of the total cost (95.45%). Healthcare cost was found to be the second highest share representing 4370.1 million baht (2.7% of the total cost). The number of premature death attributable to alcohol consumption in 2021 was estimated at 22,804.

**Conclusions:**

Alcohol continues to impose a substantial economic burden in Thailand. Enforcement of existing well-formulated alcohol control policies is urgently required to mitigate the economic impact of alcohol consumption in the country.

## Background

In 2016, alcohol drinking was the 7th leading risk factor for death and disability-adjusted life year (DALY) globally [1]. Although commonly viewed as personal choice alcohol consumption not only affects the health of individual drinkers but also contributes to productivity loss as well as many social problems such as drunk driving, crime, and domestic violence. As a result, existing evidence consistently indicated that alcohol was one of the key risk factors contributing to avoidable economic loss worldwide [2–4].

The estimates from the economic cost of alcohol consumption studies provide essential information for several purposes: to raise public awareness of the burden of alcohol on society, to guide policy decision making and public health planning for alcohol control initiatives, to justify budgets for alcohol-related policies, and to provide baseline measures to determine the efficacy of the alcohol policy or program. Based on the recent systematic review [2], the average cost of alcohol use per adult after adjusting for the omission of cost components in the analysis was estimated at 1306 Int$ or accounted for 2.6% of Gross Domestic Product (GDP). Notably, of the 29 studies included in the review, 25 were conducted in high-income countries. Given that the economic impact of alcohol drinking depends on various factors including prevalence and pattern of drinking, economic status, and characteristics of population and health system [5], country-specific studies on economic impact of alcohol consumption are necessary.

Thailand is a middle-income country located in mainland South-East Asia. According to the National Health Examination Survey (NHES), the prevalence of alcohol drinking in Thailand decreased from 72.6% in 2003/4 [6] to 55.9% in 2014 [7] in males and 35.1–23.0% in females. However, the prevalence in 2019/2020 was slightly increased to 59.0% and 31.0% in males, and females, respectively [8]. In 2021, the excise tax from alcoholic beverages in Thailand was 140,667 Million baht, representing 0.87% of the country’s GDP [9]. It should be noted that in 2014, alcohol was identified as the primary risk factor for attributable DALYs in males, causing 12% of DALY losses in the country [10]. To date, studies on the economic costs of alcohol consumption in Thailand have been conducted in 2006 [11], 2011 [12], and 2017 [13]. These studies showed that the economic burden of alcohol ranged between 0.56 and 1.99% of Thailand’s GDP. Due to the changes in the prevalence of drinking and epidemiological changes in alcohol-related diseases along with its costs, an update of the estimates of alcohol-attributable costs is needed to reflect the current situation for supporting evidence-based public health policy decision making in the country. Therefore, the objective of this study was to estimate the economic cost of alcohol consumption in Thailand, in 2021.

## Methods

### Study design and sample

To estimate gross costs of alcohol consumption in Thailand, 2021, a prevalence-based cost-of-illness methodology was employed. Cost-of-illness, also known as economic cost-of-illness or economic cost [14], is a summary of the costs of a particular disease or condition to society [15]. Cost-of-illness aimed to measure the economic burden of the disease or condition imposed on society [14, 16]. The terms economic burden and economic costs are frequently used interchangeably.

A societal perspective was adopted for the analysis. Both direct and indirect costs were estimated. All costs were presented in Thai baht, 2021 (36.14 baht = 1 US$). Then, the estimates were displayed as a percentage of the country’s GDP to facilitate the cross-country comparison without the intention to measure the impact of drinking on the growth of the economy.

In this study, 25 diseases/conditions attributable to alcohol identified by the World Health Organization (WHO) Global Burden of Disease 2020 [17, 18] were included in the analysis. To estimate the number of patients and deaths attributable to alcohol consumption, alcohol-attributable fractions (AAFs) were calculated for each disease, using the following formula [19]:

$$AAF_{i} { }\left( {\text{ \% }} \right){ } = \frac{{\mathop \sum \nolimits_{j = 1}^{k} P_{j} *(RR_{ij} - 1)}}{{\mathop \sum \nolimits_{j = 1}^{k} P_{j} * \left( {RR_{ij} - 1} \right) + 1}}*100$$

where P_j_ = Prevalence of alcohol consumption at drinking level *j*, RR_ij_ = Relative risk (RR) of developing alcohol-related disease *i* at drinking level *j* compared to non-drinker.

In this analysis, the prevalence of alcohol consumption by drinking categories was adopted from the previous study [13], which was estimated from the Smoking and Drinking Behavior Survey 2017 [20]. The drinking categories were classified based on consumption of pure alcohol, measured in grams per day into (1) light alcohol drinking (female > 0 −  < 20 g/day, male > 0 −  < 40 g/day), (2) moderate alcohol drinking (female 20 – 40 g/day, male 40 – 60 g/day), (3) heavy alcohol drinking (female > 40.0 g/day, male > 60.0 g/day) [21]. Information on the RRs of diseases caused by alcohol consumption was derived from the relevant meta-analyses [11, 17, 22–25]. The AAFs estimated in this study are displayed in Table 1.

**Table 1:** The Alcohol-attributable Fraction (AAF) used in the study

	Disease	AAF (%)		Sources of relative risk (RR)
		Male	Female	
1	Tuberculosis	28	4	Rehm et al. [17]
2	HIV/AIDS	4	0	Rehm et al. [22]
3	Lower respiratory infections: Pneumonia	6	1	Rehm et al. [17]
4	Lip and oral cavity cancers	36	5	Rehm et al. [17]
5	Other pharyngeal cancers	29	5	Bagnardi et al. [23]
6	Esophagus cancer	31	8	Rehm et al. [17]
7	Colon and rectum cancers	9	1	Rehm et al. [17]
8	Liver cancer	12	1	Rehm et al. [17]
9	Breast cancer	0	2	Rehm et al. [17]
10	Larynx cancer	21	3	Rehm et al. [17]
11	Diabetes mellitus	− 11	− 3	Rehm et al. [17]
12	Alcohol use disorders	100	100	Rehm et al. [17]
13	Epilepsy	19	2	Rehm et al. [17]
14	Hypertensive heart disease	14	− 5	Rehm et al. [17]
15	Ischemic heart disease	− 10	1	Rehm et al. [17]
16	Ischemic stroke	5	− 3	Rehm et al. [17]
17	Hemorrhagic stroke	10	3	Rehm et al. [17]
18	Cardiomyopathy, myocarditis, endocarditis	9	3	Mathey et al. [24]
19	Cirrhosis of the liver	46	22	Rehm et al. [17]
20	Pancreatitis	27	− 2	Rehm et al. [17]
21	Road injury^a^	29	29	The Secretariat of the Prime Minister 2021 [25]
22	Drowning	12	12	Thavorncharoensap et al. [11]
23	Exposure to mechanical forces	73	73	Thavorncharoensap et al. [11]
24	Self-harm	23	23	Thavorncharoensap et al. [11]
25	Interpersonal violence	57	57	Thavorncharoensap et al. [11]
26	Offences against sexuality, body and life	31	31	Laixuthai et al. [35]
27	Offences against Property	3	3	Laixuthai et al. [35]
28	Offences relating to criminal damage	7	7	Laixuthai et al. [35]
29	Road traffic accident	33.7	33.7	Office of the National Economic and Social Development Council 2021 [37, 38]

^a^the proportion of road injuries attributable to alcohol during New Year festival, 2021

### Cost estimates

#### Direct costs

In this study, direct care costs included healthcare costs, costs of law enforcement, and cost of property damage due to road traffic accidents.

##### Healthcare costs

Healthcare costs consisted of the cost incurred in the out-patient department (OPD) and the cost incurred in the in-patient department (IPD). Healthcare cost attributable to alcohol was estimated as the product of the number of alcohol-attributable patients and the disease-specific unit cost of treatment. To determine the number of patients attributable to alcohol in 2021, the number of patients with each disease in 2021 was multiplied by the corresponding alcohol-attributable fraction (AAF). The total number of patients for a particular disease and the disease-specific unit cost of treatment in 2021 were derived from the database of National Health Security Office (NHSO), according to the ICD-10 codes. This database covered information on patients under the Universal Health Coverage (UHC) scheme in Thailand. The assumption that the total number of patients covered under the UHC program represented 70% of the overall patient population in the country was employed to estimate the total number of patients in Thailand.

##### Costs of law enforcement

In this analysis, the costs of law enforcement attributable to alcohol consumption included justice system costs incurred at police stations, court of justice, office of the attorney general, and prison. The costs were calculated by multiplying the number of crimes and offenses attributable to alcohol consumption with the unit cost per case of each state agency.

To estimate the unit cost per case, the annual budget related to the justice work allocated to each agency by the Budget Bureau in 2021 [26–30] was divided by the number of crimes and offenses prosecuted by the agency in the same year. The number of prosecuted crimes and offenses in 2021 was obtained from the annual report of the following agencies: the Royal Thai Police, the Court of Justice, the Office of the Attorney General, and the Department of Corrections, Ministry of Justice [31–34]. On the other hand, the proportion of crimes and offenses attributable to alcohol consumption was derived from the previous study in Thailand [35], as shown in Table 1.

##### Cost of property damage due to road traffic accidents

In this study, the cost of property damage due to road traffic accidents attributable to alcohol consumption was estimated by multiplying the total cost of property damage due to road traffic accidents in 2021, which was reported by the Royal Thai Police [36] with the proportion of road traffic accidents attributable to alcohol during New Year and Songkran festival in Thailand, 2021, which reported by the Office of the National Economic and Social Development Council [37, 38].

#### Indirect costs

Indirect costs included in the analysis were cost of premature mortality and absenteeism from out-patient hospital visits and hospitalization.

##### Cost of premature mortality

In this analysis, premature mortality was defined as death that occurs before the expected life expectancy of the Thai population. Human capital approach was adopted to estimate the cost of productivity loss due to premature mortality. To estimate the number of alcohol-attributable deaths for each disease, the total number of deaths by age and gender for each disease was multiplied by the corresponding disease-specific AAF. Information on the number of deaths by age and gender was derived from the Strategy and Planning Division, Office of the Permanent Secretary, Ministry of Public Health [39] and was adjusted by the proportion of unidentified causes of death based on the recent study in Thailand [40]. The cost of premature mortality was, then, estimated by multiplying the number of alcohol-attributable deaths, by age and gender with the present value of lost earnings by age and gender. As recommended by the Guideline for Health Technology Assessment in Thailand, Gross National Income (GNI) per capita in 2021 (232,176 baht) [41] was used. The growth rate of 2.94% per year, which was the average growth rate between 2000 and 2020 was applied [42]. The number of years lived beyond each certain age up to the life expectancy was obtained from the WHO life table for Thailand [43]. To convert the future cost into the present value, the discount rate of 3% was adopted [44].

##### Costs of hospital-related absenteeism

Costs of absenteeism from out-patient hospital visits and hospitalization attributable to alcohol consumption were estimated by multiplying the number of patients attributable to alcohol by the average number of absent days from out-patient visits and hospitalization per patient by the daily wage. The information on the annual number of disease-specific out-patient visits and in-patient length of stay per patient in 2021 was derived from the database of NHSO, which contained ICD-10 codes for each hospital visit. For our study, one out-patient visit was set to a half day loss. Meanwhile, the number of absent days due to hospitalization was assumed to be equal to the length of stay.

### Sensitivity analysis

One-way sensitivity analyses were performed to examine the extent to which the results are affected by the choice of parameters and method used in the estimations. In the analyses, discount rates of 0% and 6% were used, as recommended by the Thai Guideline for Health Technology Assessment [45]. The alteration of the prevalence of alcohol consumption (± 10%, ± 20%) and the use of net estimation approach was also examined in the analyses. In addition, the alternative assumptions used in evaluating productivity loss were also explored. At present, Thailand’s official retirement age is 60 years old. Nevertheless, data from Thailand’s Labor Force Survey 2022 [46] shows that slightly less than 50% of Thais aged 60 years and over are in the labor market. In the sensitivity analyses, we, therefore, assumed a 50% drop in income after aged 60 years old. In the analyses, we also assumed that there was no productivity loss after the age of 65 years old, as there is a proposal to extend the retirement age in the country from 60 to 65 years.

## Results

As summarized in Table 2, the economic cost of alcohol consumption in Thailand for the year 2021 was calculated at 165,450.5 million baht. This was equivalent to 1.02% of Thailand GDP’s and accounted for 2500 baht per capita. Indirect costs made up the largest proportion of the total cost (96.32%) with an estimate of 159,358.8 million baht while direct costs were evaluated at 6091.7 million baht.

**Table 2:** Summary of the economic cost of alcohol consumption in Thailand, 2021

Cost category	Economic cost (million baht)	Percentage (%)
**Direct costs**	**6,091.68**	**3.68**
Health care cost		
Total	4370.13	2.64
Out-patient	1422.25	0.86
In-patient	2947.88	1.78
Cost of law enforcement		
Total	1704.34	1.03
Police	144.44	0.09
Court	519.68	0.31
Attorney	275.00	0.17
Prison	765.22	0.46
Cost of property damage	17.21	0.01
**Indirect costs**	**159,358.77**	**96.32**
Cost of premature mortality	157,918.66	95.45
Cost of absenteeism	1440.12	0.87
Total cost	165,450.45	100.00

For the indirect costs, the cost of premature mortality was found to be the major cost component, representing 95.45% of the total cost with estimates of 157,918.7 million baht (134,424.7 million baht in males and 23,493.9 million baht in females). In 2021, premature death attributable to alcohol consumption’s number was estimated at 22,804 (male = 19,678, female = 3127), resulting in 666,393 years of life loss. As shown in Table 3, the five leading causes of premature mortality costs were road injuries (47,821.9 million baht), followed by cirrhosis (23,740.0 million baht), self-harm (12,944.3 million baht), alcohol use disorder (11,535.6 million baht), and tuberculosis (9873.9 million baht), respectively. When focusing on morbidity-related productivity loss, cost of absenteeism was estimated at 1440.1 million baht (0.87% of the total cost).

**Table 3:** Health care cost, cost of hospital-related absenteeism, and cost of premature mortality attributable to alcohol in Thailand, 2021, by disease and gender

	Disease	Health care cost		Cost of absenteeism		Cost of premature mortality		Total cost	
		(Million baht)		(Million baht)		(Million baht)		(Million baht)	
		Male	Female	Male	Female	Male	Female	Male	Female
1	Tuberculosis	240.29	12.37	108.59	5.59	9370.54	503.40	9719.43	521.36
2	HIV/AIDS	87.01	9.57	14.80	1.49	820.02	60.46	921.82	71.52
3	Lower respiratory infections: Pneumonia	1268.74	165.68	232.95	30.32	6265.21	478.10	7766.89	674.10
4	Lip and oral cavity cancers	85.91	8.57	20.96	2.03	2472.92	156.91	2579.79	167.51
5	Other pharyngeal cancers	60.45	1.52	14.07	0.33	1087.87	20.17	1162.39	22.02
6	Esophagus cancer	90.81	3.78	20.55	0.84	3036.55	94.99	3147.91	99.61
7	Colon and rectum cancers	152.06	15.09	26.54	2.65	1334.38	130.06	1512.98	147.80
8	Liver cancer	142.39	3.37	19.38	0.46	7748.74	156.02	7910.51	159.85
9	Breast cancer	−	23.39	−	5.23	−	607.95	−	636.57
10	Larynx cancer	29.68	0.32	7.08	0.07	870.57	6.38	907.32	6.78
11	Diabetes mellitus	−	−	−	−	−	−	−	−
12	Alcohol use disorders	413.76	49.43	290.11	34.63	10,387.47	1148.14	11091.34	1232.20
13	Epilepsy	128.81	7.42	38.69	2.26	835.70	48.77	1003.20	58.44
14	Hypertensive heart disease	686.51	−	357.35	−	2303.25	−	3347.12	−
15	Ischemic heart disease	−	27.36	−	3.40	−	477.04	−	507.80
16	Ischemic stroke	152.78	−	54.72	−	2722.73	−	2930.22	−
17	Hemorrhagic stroke	74.88	15.56	18.88	3.92	8380.68	1257.70	8474.43	1277.18
18	Cardiomyopathy, myocarditis, endocarditis	36.23	6.64	7.48	1.39	608.38	139.83	6.08	147.86
19	Cirrhosis of the liver	235.11	45.60	69.44	13.60	20544.89	3195.11	2849.43	3254.31
20	Pancreatitis	83.02	−	23.66	−	956.20	−	1062.88	−
21	Road injury	1.63	0.98	0.86	0.52	36,834.11	10987.87	36,836.60	10,989.37
22	Drowning	0.02	0.00	0.01	0.00	2320.67	477.16	2320.70	477.17
23	Exposure to mechanical forces	1.85	0.85	3.32	1.52	1033.94	188.59	1039.1	190.96
24	Self-harm	0.03	0.03	0.02	0.02	10,453.54	2490.77	10,453.58	2490.82
25	Interpersonal violence	0.26	0.38	0.17	0.24	4036.37	868.54	4036.80	869.16
	Total	3972.21	397.92	1329.62	110.50	13,4424.70	23,493.95	139,726.53	24,002.38
		4370.13		1440.12		157,918.66		163,728.91	

Among the direct costs which were estimated at 6091.7 million baht (3.7% of the total cost), healthcare cost was estimated at 4370.1 million baht (2.7% of the total cost) while justice system cost was estimated at 1704.3 million baht (1.03% of the total cost), and cost of property damage was estimated at 17.2 million baht (0.01% of the total cost).

Healthcare costs attributable to alcohol drinking in Thailand in 2021 by gender and disease are also shown in Table 3. Our analysis found that the five leading causes of healthcare cost were lower respiratory tract infection (1434.4 million baht), followed by hypertensive heart disease (686.5 million baht), alcohol disorder (463.2 million baht), cirrhosis (280.7 million baht), and tuberculosis (252.7 million baht), respectively.

As for the costs of law enforcement, the cost incurred at prison represented the largest proportion (765.2 million baht), followed by the costs incurred at court of justice (519.7 million baht), and the office of attorney general (275.0 million baht), respectively.

As shown in Table 4, the estimates of sensitivity analyses ranged between 81,148.1 million baht (− 50.95% change from baseline) and 299,487.8 million baht (+ 81.01% change from baseline). The choice of discount rate had the highest impact on the result, followed by the assumption of no productivity loss beyond the age of 65 years old.

**Table 4:** Sensitivity analyses

Parameter/method	Economic cost (million baht)	% change
Base case (Gross cost estimation)	165,450.45	NA
Net cost estimation	150,026.08	− 9.32
Prevalence increases from base case 10%	171,504.43	+ 3.66
Prevalence increases from base case 20%	177,327.22	+ 7.18
Prevalence decreases from base case 10%	159,143.23	− 3.81
Prevalence decreases from base case 20%	152,557.14	− 7.79
Discount rate at 0%	299,487.76	+ 81.01
Discount rate at 6%	108,499.55	− 34.42
No productivity loss beyond age of 65	81,148.10	− 50.95
50% reduction of the earning after the age of 60	113,546.47	− 31.37

*NA* Not applicable

## Discussion

Alcohol consumption incurred substantial economic impacts on Thai society, representing 1.02% of Thailand’s GDP. Indirect costs accounted for the largest proportion, representing 96.32% of the total cost. Our estimate was lower than that of the recent systematic review [2], which indicated that the costs of alcohol use were equal to 2.6% of the GDP but were consistent in that the indirect costs were the major share of the total cost. This could be partly explained by the fact that the estimates from the previous study [2] were adjusted for the omission of cost components in the analysis.

As compared to the estimates from the previous study in the country, which was conducted in 2006 [11], this recent estimate was lower (1.99% vs 1.02% of the GDP). This is probably due to the lower prevalence of drinking in this analysis. In this study, the proportion of current drinkers was 47.5% and 10.6% in males, and females respectively. Albeit, the proportion of current drinkers used in the previous study [11] was 72.6% and 35.1% in males and females, respectively. Furthermore, it is worth noting that the methodology, cost components, and source of information used in this study and previous studies in the country [11–13] were slightly different. For example, the previous study in 2006 [11] included the presenteeism costs while the others did not. In addition, the source of income and assumption used in the estimation of indirect costs varied across studies. Therefore, a direct comparison of the findings across the studies should be carefully considered.

It should be noted that there is no consensus on the latency period between alcohol consumption and the year of disease occurrence. Based on the available evidence [47], the time to the first effect varied from immediately to 10 years while the time to get the full effect ranged from 5 to 20 years, depending on the type of disease. Due to the availability of data, the lag time of 4 years was adopted in this study. Given that the time to get the full effect ranged from 5 to 20 years, the use of 4 year-lag time in our analysis might result in the overestimation of the cost.

Although our findings indicated that alcohol imposed a substantial economic burden on society, this analysis was more likely to be underestimated due to several reasons: (1) some of the diseases were not included in the analysis, such as depression, low-birth weight, ethanol toxicity, (2) cost of productivity loss due to disability resulted from road traffic accidents was not included, (3) absenteeism cost only included cost due to hospital-related absence, (4) presenteeism cost, informal care cost, and intangible cost were not included, and (5) the costs were estimated during the COVID-19 pandemic period when healthcare utilization [48], the number of road traffic accidents, and crimes were expected to be decline due to the COVID-19 related restrictions policy.

This study has some limitations. Firstly, the proportion of road traffic accidents/road injuries attributable to alcohol consumption may be overestimated using statistics during the festival period. Furthermore, the proportion of alcohol-related crimes and offenses was derived from the previous local study conducted in only one province in 2001.

Finally, the implementation of effective policies and interventions that aim to reduce the prevalence of drinking or to mitigate the negative effects of alcohol consumption should be prioritized to decrease the economic burden associated with alcohol consumption in the country. To date, various effective alcohol policies [49] such as taxation, restriction of alcohol marketing, restriction on alcohol availability (i.e. restriction on minimum purchase age of the buyer, time of sale of alcohol, and alcohol outlet location), and drunk-driving laws have been implemented for many years in Thailand [50, 51]. Nevertheless, a slightly increase in alcohol consumption was yet observed recently [50]. A recent study suggested that the lack of robustness of law and policy enforcement could hamper the effectiveness of such policies [52]. Therefore, well-formulated law enforcement strategies on alcohol control policy are highly warranted.

## Conclusions

Alcohol continues to impose a substantial economic burden on Thai society. Hence, the government and related organizations need to put priority on the policies aims at reducing alcohol consumption, including policies to mitigate the negative consequences of alcohol drinking. Furthermore, law enforcement on alcohol control policy should be emphasized to reduce the economic burden of alcohol consumption in the country.

## Data Availability

The datasets generated during and/or analyzed during the current study are available from the corresponding author on reasonable request.

## Abbreviations

AAFs: Alcohol-attributable fractions
DALY: Disability-adjusted life year
GDP: Gross domestic product
GNI: Gross national income
IPD: In-patient department
NHES: National Health Examination Survey
NHSO: National Health Security Office
OPD: Out-patient department
RR: Relative risk
UHC: Universal Health Coverage
WHO: World Health Organization
